# Une manifestation exceptionnelle de la maladie de Behçet: la thrombose du ventricule gauche (à propos d’un cas)

**DOI:** 10.11604/pamj.2023.44.200.12844

**Published:** 2023-04-26

**Authors:** Mehdi Berrajaa, Hicham El Meghraoui, Mohamed Ofkire, Noha El Ouafi

**Affiliations:** 1 Faculté de Médecine et de Pharmacie Oujda, Service de Cardiologie, CHU Mohammed VI, Oujda, Maroc

**Keywords:** Behçet, thrombose, anévrysme, aphthose, cas clinique, Behçet, thrombosis, aneurysm, aphthous ulcers, case report

## Abstract

Dans la maladie de Behçet, la survenue de thrombose intracardiaque est rare, exceptionnellement révélatrice. Nous rapportons le cas d´un patient âgé de 48 ans, suivi pour un érythème noueux, admis pour prise en charge d´un accident vasculaire cérébral (AVC). L´interrogatoire objective une aphtose buccale récurrente. L´examen cardiovasculaire était sans particularité, imagerie par résonance magnétique (l´IRM) objectivait un AVC pariétal postérieur gauche. Une échocardiographie trans-thoracique a été réalisée objectivant un anévrysme apical totalement thrombosé mesurant 42mm/36mm; un scanner du corps entier à la recherche d´autres localisations était sans particularité en dehors de l´atteinte cardiaque. Le diagnostic de la maladie de Behçet a été retenu devant l´association d´aphtose buccale récurrente, de l´érythème noueux et de l´anévrysme thrombosé du ventricule gauche. L´évolution était favorable sous traitement médical associant la corticothérapie, l´anticoagulation et l´immunosuppression. La découverte de thrombus intracardiaque ou d´anévrysme ventriculaire chez un jeune homme, résident en région d´endémie devrait faire suspecter la maladie de Behçet, dont le pronostic est principalement en relation avec la gravité de l´atteinte cardiovasculaire.

## Abstract (en)

Intracardiac thrombus is an exceptional complication revealing Behçet’s disease. We here report the case of a 48-year-old patient admitted with stroke. Clinical examination showed recurrent oral aphthous ulcers. Cardiovascular examination was normal, magnetic resonance imaging (MRI) objectified left posterior parietal stroke. Transthoracic echocardiogram showed totally thrombosed apical aneurysm measuring 42mm/36mm. Total-body computed tomography (CT) scan was performed to detect other abnormalities. It was normal apart from cardiac involvement. The diagnosis of Behçet’s disease was made based on oral aphthous ulcers, polyarteritis nodosa and thrombosed left ventricular aneurysm. Outcome was favorable under medical treatment combining anticoagulants, corticosteroids and immunosuppression.

## Introduction

La maladie de Behçet est une vascularite multisystémique dont l´étiologie toujours non élucidée. Cette maladie se présente cliniquement sous forme d´uvéite, d´ulcérations buccales et génitales, avec possibilité de manifestations neurologiques, cutanées et articulaires. Des thromboses artérielles ou veineuses peuvent également survenir. L´atteinte cardiaque, notamment la formation de thrombus intracardiaque, est exceptionnelle. Nous rapportons le cas d´un patient atteint de maladie de Behçet et qui a été révélée par la découverte d´un thrombus intra-ventriculaire gauche chez un patient jeune.

## Patient et observation

**Informations relatives aux patients (présentation du patient):** il s´agit d´un patient âgé de 48 ans, diabétique depuis 2 ans sous sulfamides, suivi depuis 1987 pour un érythème noueux sous corticothérapie. À l´interrogatoire le patient rapporte la notion d´aphtose buccale récurrente.

**Résultats cliniques:** l´examen cardiovasculaire était sans particularités. L´examen neurologique retrouve une hémiparésie droite sans participation faciale.

**Chronologie:** l´histoire de la maladie a commencé le jour même de son admission, par l´installation brutale d´un déficit moteur motivant le patient à consulter au service des urgences.

**Figure 1:**
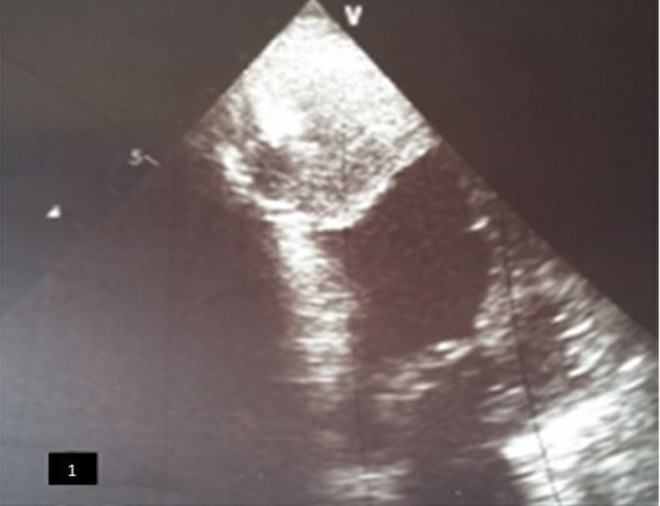
échographie trans-thoracique en fenêtre apical quatre cavités montant un énorme thrombus apical

**Figure 2:**
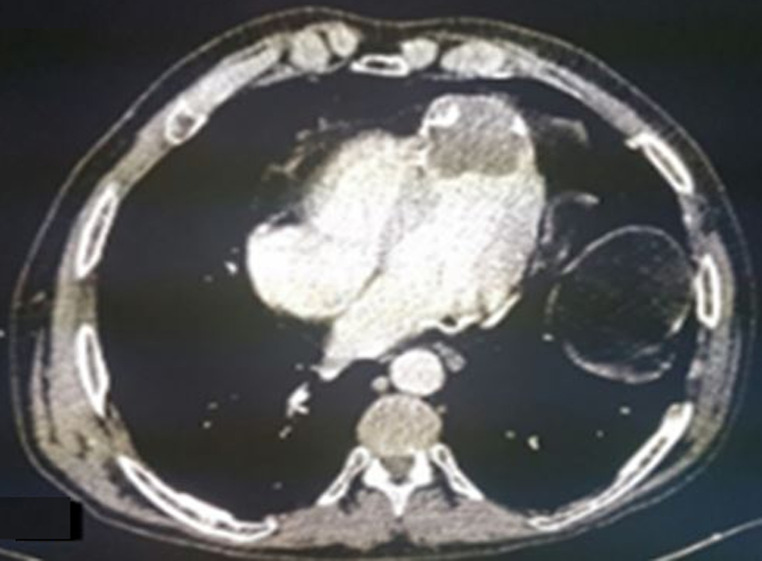
coupe tomodensitométrique axiale objectivant un apex de paroi amincis, anévrysmale, siège d´un thrombus hypodense

**Figure 3:**
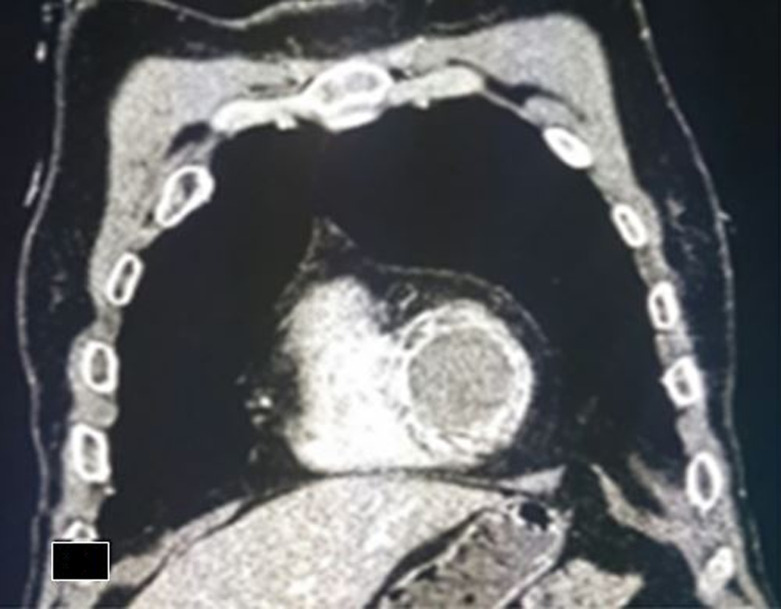
coupe tomodensitométrique coronale objectivant la coque calcifiée du thrombus

**Figure 4:**
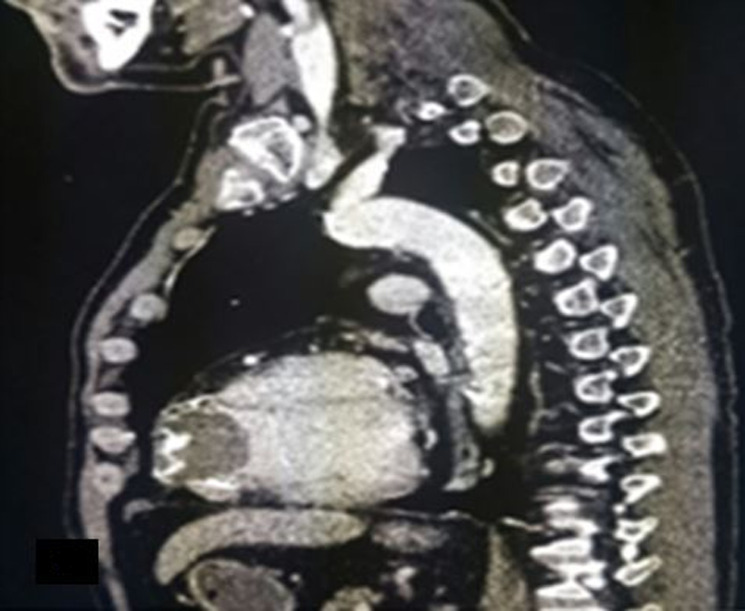
coupe tomodensitométrique sagittale montrant l´anévrysme thrombosé du ventricule gauche

**Démarche diagnostique:** le scanner cérébral était sans anomalie, l´IRM a objectivé un AVC pariétal postérieur gauche séquellaire. L'électrocardiogramme (ECG) s´inscrit en rythme sinusal avec des ondes Q et ondes T négatives en antérieur. Une échographie trans-thoracique a été réalisée, a objectivé un anévrysme apical du ventricule gauche totalement thrombose mesurant 42 mm/36 mm (Figure 1). L´angioscanner cérébro-thoraco-abdomino-pelvien à la recherche d´autres anévrysme était sans particularités en dehors de l´atteinte cardiaque (Figure 2, Figure 3, Figure 4). Elle retrouve un ventricule gauche (VG) dont l´apex est anévrysmal de parois très amincies, siège d´une masse hypo-dense avec une coque calcifiée et ne réhaussant pas au produit de contraste. L´échographie Doppler des troncs supra-aortique et des membres inférieurs étaient sans anomalie.

Sur le plan biologique, le dosage des anticorps anti-phospholipides, anti-CCP, ANCA, AAN était négatifs ainsi que celui du facteur rhumatoïde et de la cryoglobuline. Les sérologie virales hépatitique, VIH et de la syphilis étaient négatives. Le diagnostic de la maladie de Behçet dans sa forme sévère a été retenu devant l´association de de l´apthtose buccale, de l´érytème noueux et de l´anévrysme cardiaque thrombosé.

**Intervention thérapeutique:** le patient a été mis sous corticothérapie par bolus de méthyl-prédnisolone (15mg/kg pendant trois jours, puis relais per os à 1mg/kg/j de prednisone). Une immunosuppression par cyclophosphamide et une anticoagulation par anti-vitamine K (AVK) ont été également instaurées.

**Suivi et résultats:** l'évolution sous traitement a été marquée par une régression de la taille de l´anévrysme et du thrombus.

**Perspective du patient:** le patient a été informé avant de démarrer le traitement médical pour lequel il était d´accord. Étant donné le caractère emboligène du thrombus; le traitement chirurgical lui a été proposé, non réalisé pour la réticence du patient et de sa famille.

**Consentement éclairé:** le patient a exprimé son consentement pour la publication de ce «case report» accompagné des figures.

## Discussion

Au cours de la maladie de Behçet, l´atteinte vasculaire est fréquente et survient en particulier souvent chez les patients de sexe masculin jeunes, fréquemment sans autres facteurs de risque comme le tabagisme [1,2]. L'atteinte cardiaque secondaire à la maladie de Behçet est exceptionnelle, et ne survient que chez 6% des patients atteints de cette maladie [3]. L´atteinte cardiaque lors de la maladie de Behçet peut impliquer différentes tuniques cardiaques. Les types d´atteintes cardiaques rapportés dans la littérature regroupent les péricardites représentant 29% des formes d´atteintes cardiaques, les atteintes de l´endocarde sous forme de thromboses intracardiaques dans 29% des cas, d´insuffisance aortique survenant dans 25% des cas. Les atteintes myocardiques peuvent se révéler sous forme d´infarctus du myocarde survenant dans 15% des cas, de fibrose myocardique observée dans 8% des cas, de myocardites, et d´anévrismes du ventricule gauche [4].

Les thrombi intracardiaques secondaires à la maladie de Behçet touchent souvent le cœur droit et sont fréquemment observés en association avec des thromboses veineuses à type d´embolie pulmonaire (60%). Une extension du matériel thrombotique vers la veine cave inférieure est rapportée dans 40% des cas [5].

L´imagerie, en particulier l´échographie cardiaques, permet de poser le diagnostic des thrombi intracardiaques en révélant une masse hétérogène intracardiaque d´échogénicité supérieure à celle du sang, et qui adhère, avec une large base d´implantation à la paroi cardiaque.

Le thrombus intracardiaque peut être confondu avec des tumeurs primitives du cœur, notamment les myxomes. Ces derniers prennent l´aspect de masse ovalaire ou sphérique, s´implantant sur une base étroite et sont souvent localisées au niveau de la fosse ovale. Dans les cas de fibrose endomyocardique secondaire à la maladie de Behçet, un aspect pseudo-tumoral est rarement rapporté avec un aspect hyperéchogène de l´endocarde, prêtant ainsi confusion avec un thrombus intracardiaque [6].

Le thrombus intracardiaque est fréquemment observé en association avec une atteinte vasculaire pulmonaire notamment à type d'anévrysme de l´artère pulmonaire. Il s´agit d´une complication artérielle particulièrement grave qui se manifeste par des douleurs thoraciques, une dyspnée et des hémoptysies (93%) pouvant parfois être cataclysmiques (26%) et mortelles.

Classiquement, le thrombus intracardiaque secondaire à la maladie de Behçet est adhérent à la paroi, expliquant ainsi sa nature peu emboligène. Néanmoins, des emboles secondaires à des thrombi intracardiaques dans le cadre de maladie de Behçet ont été rapportés [7,8]. L'association d´un thrombus intracardiaque avec une thrombose veineuse est observée dans 56% des cas [9]. Le mécanisme de formation de thrombi intracardiaques chez les patients atteints de maladie de Behçet n´est pas entièrement élucidé mais peut faire impliquer des lésions ischémiques endothéliales qui favoriseraient une agrégation plaquettaire anormale [10]. Dans le même cadre de maladie de Behçet, l´association à un syndrome des anticorps anti-phospholipides ou à une thrombophilie associée pourrait expliquer la survenue de thrombose [11,12]. Le traitement de la thrombose intracardiaque secondaire à la maladie de Behçet n'est pas encore codifié et la conduite thérapeutique devra être alors décidée au cas par cas.

Plusieurs études [4,10,13,14] ont rapporté une efficacité du traitement médical seul (antivitamine K, corticoïde, et colchicine). Dans les formes sévères, le traitement immunosuppresseur est souvent indiqué. Un traitement fibrinolytique pourra aussi être discuté vue qu´il a démontré une bonne évolution, quoique les résultats sont issus de peu d´études [14-16]. Les anticoagulants devront être utilisés avec prudence vue le risque de complications veineuses qu´elles peuvent présenter. En effet, les recommandations de l´EULAR (*European League Against Rheumatism*) ne recommandent pas de traitement anticoagulant chez ces patients, argumentant le rôle principal de l´inflammation dans la formation du thrombus intra-cardiaque, la fréquente présence d´anévrysme notamment pulmonaires ce qui prédispose à un risque hémorragique, et la nature adhérente du thrombus vis-à-vis de la paroi cardiaque ce qui explique un faible risque emboligène [17].

La chirurgie devrait être réservée aux échecs du traitement médical ou aux embolies pulmonaires massives, car il s'agit d'un geste délicat avec risque de récidive du thrombus. Les récidives peuvent évoluer favorablement après traitement médical ou après fibrinolyse [16].

## Conclusion

La découverte de complications grave à type de thrombus intracardiaque, d´anévrysme ventriculaire, chez un sujet jeune de sexe masculin, en absence de facteurs de risque cardiovasculaire, en région d´endémie devrait faire suspecter la maladie de Behçet. Notre cas clinique, a permis de mettre en valeur la place de l´échocardiographie dans le diagnostic de ces atteintes cardiovasculaires, qui conditionnent le pronostic. Le traitement médical est toujours de mise, reposant essentiellement sur l´immunomodulation et l´immunosuppression, l´anticoagulation est controverse, la chirurgie est réservée en cas d´échec de traitement médical.
